# Supplementary figures and images for: Patterns of metformin monotherapy discontinuation and reinitiation in people with type 2 diabetes mellitus in New Zealand
Source: PLoS One. 2021 Apr 21;16(4):e0250289. doi: 10.1371/journal.pone.0250289 (PMC8059805; doi:10.1371/journal.pone.0250289)

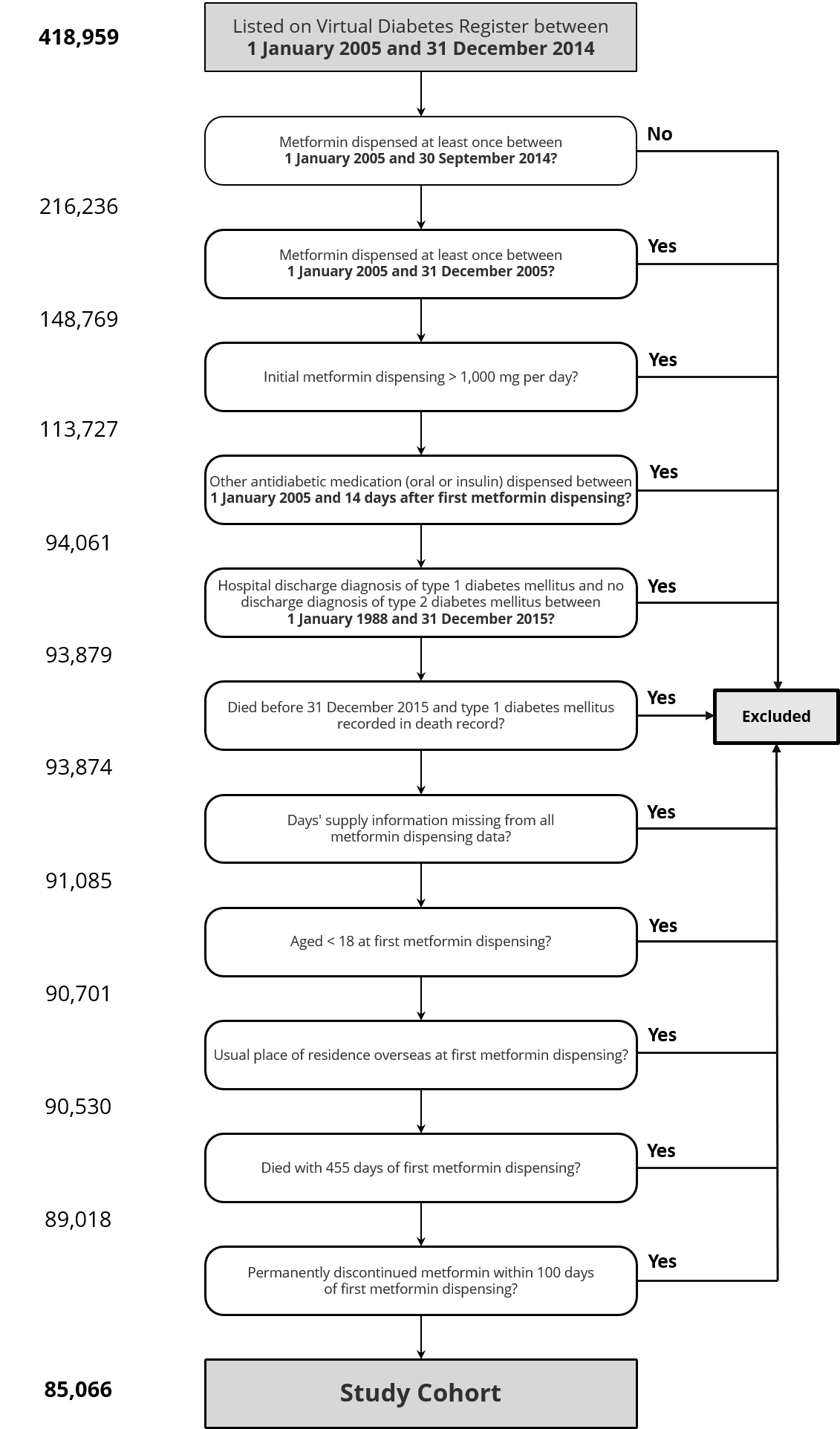

Supplement: S1 Fig — The numbers represent the people in the cohort after the previous criteria have been applied. (TIF) [file pone.0250289.s001.tif]

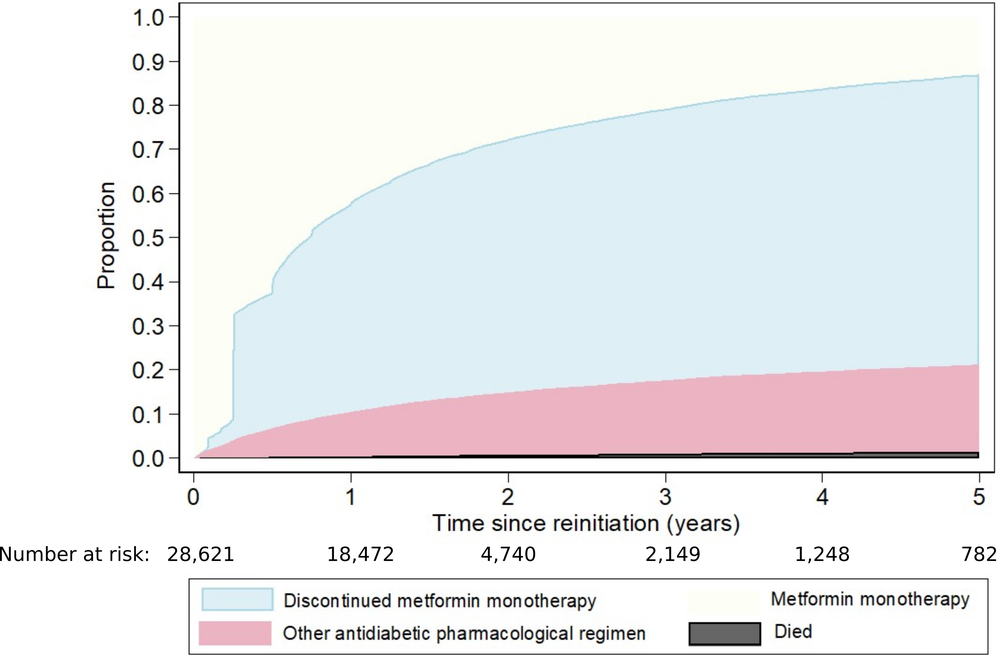

Supplement: S2 Fig — (TIF) [file pone.0250289.s002.tif]
