# Supplementary material for: Patterns of metformin monotherapy discontinuation and reinitiation in people with type 2 diabetes mellitus in New Zealand
Source: PLoS One. 2021 Apr 21;16(4):e0250289. doi: 10.1371/journal.pone.0250289 (PMC8059805; doi:10.1371/journal.pone.0250289)
Supplement: S1 Table — (DOCX) [file pone.0250289.s003.docx]

**S1 Table.** Comparison of cumulative proportions who discontinued metformin monotherapy after first reinitiation by person- and healthcare-related factors

| **Person- or healthcare-related factor** | | **Cumulative proportion (95% CI) who discontinued** | | |  | | **Hazard ratio** | | |  |
| --- | --- | --- | --- | --- | --- | --- | --- | --- | --- | --- |
|  |  | **End of year 1** | **End of year 2** | **End of year 5** | |  | | **Unadjusted (95% CI)** | **Adjusted^*^ (95% CI)** | |
| **Age at reinitiation (years)** | | | | | | | | | | |
|  | <25 | 75.0 (69.2–79.9) | 81.5 (75.9–85.9) | 84.9 (79.2–89.1) | |  | | 1.44 (1.28–1.62) | 1.36 (1.20–1.53) | |
|  | 25–34 | 65.7 (63.3–68.0) | 73.4 (71.1–75.5) | 77.0 (74.7–79.1) | |  | | 1.22 (1.15–1.30) | 1.18 (1.11–1.26) | |
|  | 35–44 | 57.2 (55.7–58.7) | 66.3 (64.9–67.8) | 72.3 (70.8–73.7) | |  | | Reference | Reference | |
|  | 45–54 | 50.7 (49.6–51.8) | 60.8 (59.6–61.9) | 67.9 (66.7–69.0) | |  | | 0.87 (0.83–0.90) | 0.90 (0.86–0.94) | |
|  | 55–64 | 42.8 (41.6–43.9) | 53.2 (51.9–54.4) | 63.0 (61.7–64.2) | |  | | 0.72 (0.69–0.75) | 0.80 (0.76–0.83) | |
|  | 65–74 | 37.3 (35.8–38.8) | 47.9 (46.3–49.5) | 58.5 (56.7–60.2) | |  | | 0.66 (0.63–0.70) | 0.77 (0.73–0.81) | |
|  | >75 | 34.5 (32.4–36.7) | 44.6 (42.2–46.9) | 58.3 (55.5–60.9) | |  | | 0.64 (0.59–0.69) | 0.80 (0.74–0.87) | |
| **Gender** | | | | | | | | | | |
|  | Female | 48.4 (47.6–49.3) | 58.6 (57.7–59.4) | 67.5 (66.5–68.4) |  | | Reference | | Reference | |
|  | Male | 46.8 (46.0–47.6) | 56.5 (55.7–57.4) | 64.4 (63.5–65.3) |  | | 0.94 (0.91–0.96) | | 0.96 (0.93–0.99) | |
| **Ethnicity (prioritised)^† ‡^** | | | | | | | | | | |
|  | Māori | 53.7 (52.4–55.0) | 63.0 (61.7–64.3) | 69.5 (68.1–70.8) |  | | 1.34 (1.28–1.39) | | 1.23 (1.17–1.28) | |
|  | Pacific | 54.6 (53.2–55.9) | 64.3 (62.9–65.6) | 71.1 (69.7–72.4) |  | | 1.38 (1.33–1.43) | | 1.22 (1.16–1.28) | |
|  | European | 41.6 (40.6–42.5) | 51.4 (50.4–52.4) | 61.2 (60.1–62.2) |  | | Reference | | Reference | |
|  | Asian (Non-Indian) | 43.3 (41.1–45.5) | 55.6 (53.2–57.8) | 65.7 (63.2–68.1) |  | | 1.08 (1.02–1.14) | | 1.00 (0.94–1.06) | |
|  | Indian | 48.8 (46.6–50.9) | 58.0 (55.8–60.1) | 66.8 (64.5–69.0) |  | | 1.17 (1.10–1.23) | | 1.06 (0.99–1.12) | |
|  | Other | 45.4 (40.6–50.2) | 57.0 (51.9–61.8) | 65.7 (60.3–70.5) |  | | 1.15 (1.02–1.29) | | 1.06 (0.94–1.19) | |
| **Socioeconomic deprivation (NZDep13) at reinitiation** | | | | | | | | | | |
|  | Quintile 1 | 44.0 (42.2–45.8) | 55.0 (53.1–56.9) | 63.7 (61.7–65.7) |  | | Reference | | Reference | |
|  | Quintile 2 | 43.4 (41.7–45.0) | 54.1 (52.4–55.8) | 62.8 (60.9–64.6) |  | | 1.06 (0.99–1.13) | | 0.98 (0.92–1.05) | |
|  | Quintile 3 | 44.9 (43.4–46.3) | 55.0 (53.5–56.5) | 65.1 (63.4–66.7) |  | | 1.05 (0.99–1.12) | | 0.99 (0.93–1.05) | |
|  | Quintile 4 | 47.1 (45.8–48.4) | 56.8 (55.4–58.1) | 65.2 (63.8–66.5) |  | | 1.13 (1.07–1.20) | | 1.02 (0.96–1.08) | |
|  | Quintile 5 | 51.4 (50.4–52.4) | 60.8 (59.8–61.8) | 68.3 (67.3–69.3) |  | | 1.31 (1.24–1.38) | | 1.03 (0.98–1.09) | |
| **District Health Board at reinitiation** | | | | | | | | | | |
|  | Auckland | 47.9 (46.2–49.5) | 58.0 (56.2–59.6) | 66.9 (65.1–68.7) |  | | Reference | | Reference | |
|  | Bay of Plenty | 44.9 (41.8–47.9) | 54.3 (51.1–57.4) | 63.3 (59.9–66.5) |  | | 0.92 (0.84–1.00) | | 0.92 (0.85–1.01) | |
|  | Canterbury | 44.3 (42.2–46.3) | 54.7 (52.5–56.8) | 63.9 (61.6–66.2) |  | | 0.93 (0.87–0.99) | | 0.99 (0.92–1.05) | |
|  | Capital and Coast | 47.5 (44.7–50.3) | 57.9 (55.0–60.7) | 64.8 (61.8–67.7) |  | | 0.98 (0.90–1.05) | | 1.00 (0.92–1.08) | |
|  | Counties Manukau | 51.0 (49.7–52.3) | 60.8 (59.5–62.1) | 68.8 (67.5–70.1) |  | | 1.06 (1.01–1.12) | | 1.00 (0.95–1.06) | |
|  | Hawkes Bay | 44.6 (41.5–47.6) | 54.7 (51.5–57.8) | 66.5 (63.1–69.7) |  | | 0.94 (0.86–1.02) | | 0.94 (0.86–1.03) | |
|  | Hutt | 46.6 (43.1–50.0) | 54.8 (51.2–58.3) | 63.0 (59.2–66.7) |  | | 0.96 (0.88–1.06) | | 0.95 (0.86–1.05) | |
|  | Lakes | 51.3 (47.1–55.3) | 61.6 (57.3–65.5) | 69.9 (65.4–73.9) |  | | 1.07 (0.96–1.18) | | 1.04 (0.93–1.16) | |
|  | MidCentral | 47.3 (43.9–50.6) | 57.7 (54.2–61.1) | 68.5 (64.6–72.1) |  | | 0.97 (0.89–1.06) | | 1.01 (0.92–1.11) | |
|  | Nelson Marlborough | 41.3 (36.8–45.7) | 52.4 (47.6–56.9) | 60.7 (55.6–65.5) |  | | 0.85 (0.75–0.96) | | 0.91 (0.80–1.04) | |
|  | Northland | 49.2 (46.1–52.2) | 59.4 (56.2–62.4) | 66.7 (63.4–69.8) |  | | 1.01 (0.93–1.10) | | 0.99 (0.91–1.08) | |
|  | South Canterbury | 42.2 (35.9–48.4) | 50.9 (44.2–57.2) | 59.8 (52.5–66.3) |  | | 0.82 (0.69–0.96) | | 0.88 (0.75–1.04) | |
|  | Southern | 41.0 (38.1–43.8) | 52.3 (49.3–55.2) | 61.2 (58.0–64.3) |  | | 0.86 (0.79–0.93) | | 0.94 (0.86–1.02) | |
|  | Tairawhiti | 50.2 (44.8–55.4) | 58.1 (52.5–63.2) | 63.4 (57.6–68.5) |  | | 0.97 (0.85–1.12) | | 0.90 (0.79–1.04) | |
|  | Taranaki | 47.3 (42.9–51.5) | 55.6 (51.1–59.8) | 64.5 (59.8–68.8) |  | | 0.98 (0.88–1.09) | | 1.03 (0.92–1.15) | |
|  | Waikato | 47.2 (44.9–49.4) | 57.3 (55.0–59.6) | 64.0 (61.5–66.3) |  | | 0.94 (0.88–1.01) | | 0.95 (0.88–1.02) | |
|  | Wairarapa | 46.6 (40.0–52.8) | 54.1 (47.3–60.4) | 60.3 (53.0–66.8) |  | | 0.89 (0.75–1.05) | | 0.97 (0.82–1.15) | |
|  | Waitemata | 48.3 (46.6–50.0) | 58.2 (56.5–59.9) | 66.3 (64.4–68.1) |  | | 0.98 (0.93–1.04) | | 1.01 (0.95–1.07) | |
|  | West Coast | 42.0 (32.3–51.4) | 54.1 (43.4–63.5) | 61.7 (50.4–71.1) |  | | 0.82 (0.65–1.04) | | 0.89 (0.70–1.14) | |
|  | Whanganui | 48.3 (43.3–53.1) | 55.7 (50.6–60.5) | 64.4 (58.9–69.3) |  | | 0.94 (0.83–1.07) | | 0.95 (0.84–1.09) | |
| **Charlson comorbidity index at reinitiation** | | | | | | | | | | |
|  | 0 | 48.6 (47.9–49.2) | 58.5 (57.9–59.2) | 66.8 (66.1–67.5) |  | | Reference | | Reference | |
|  | 1 | 41.4 (39.3–43.4) | 51.2 (49.1–53.4) | 60.6 (58.3–62.8) |  | | 0.88 (0.83–0.93) | | 0.99 (0.93–1.06) | |
|  | 2 | 39.3 (35.5–43.0) | 49.1 (45.2–53.0) | 58.5 (54.1–62.5) |  | | 0.86 (0.77–0.96) | | 1.05 (0.93–1.19) | |
|  | >3 | 32.4 (27.0–37.9) | 40.3 (34.3–46.1) | 49.5 (42.8–55.9) |  | | 0.67 (0.55–0.83) | | 0.89 (0.71–1.11) | |
| **History of cardiovascular disease at reinitiation** | | | | | | | | | | |
|  | No | 50.3 (49.6–51.0) | 60.3 (59.6–61.0) | 68.2 (67.5–68.9) |  | | Reference | | Reference | |
|  | Yes | 37.7 (36.5–39.0) | 47.5 (46.1–48.8) | 57.5 (56.0–58.9) |  | | 0.76 (0.73–0.79) | | 0.87 (0.82–0.91) | |
| **Cancer registration in the year before reinitiation** | | | | | | | | | | |
|  | No | 47.6 (47.0–48.2) | 57.6 (56.9–58.2) | 66.0 (65.3–66.6) |  | | Reference | | Reference | |
|  | Yes | 41.9 (34.6–48.9) | 49.4 (41.8–56.5) | 56.0 (47.8–63.5) |  | | 0.96 (0.76–1.20) | | 0.98 (0.92–1.05) | |
| **Number of hospitalisations in the year before reinitiation** | | | | | | | | | | |
|  | 0 | 48.3 (47.6–49.0) | 58.4 (57.7–59.0) | 66.6 (65.9–67.3) |  | | Reference | | Reference | |
|  | 1 | 45.1 (43.4–46.8) | 54.3 (52.5–56.0) | 62.9 (61.0–64.8) |  | | 1.03 (0.98–1.08) | | 1.07 (1.02–1.12) | |
|  | 2–4 | 44.2 (41.6–46.7) | 54.7 (52.0–57.2) | 63.6 (60.8–66.3) |  | | 0.75 (0.31–1.85) | | 0.90 (0.38–2.09) | |
|  | 5–9 | 35.3 (28.1–42.5) | 40.3 (32.7–47.7) | 49.0 (40.1–57.2) |  | | 0.95 (0.89–1.02) | | 1.02 (0.94–1.10) | |
|  | >10 | 35.6 (19.5–52.1) | 39.1 (22.2–55.6) | 51.4 (30.5–68.9) |  | | 0.92 (0.71–1.20) | | 1.14 (0.88–1.48) | |
| **Depression in the 6 months before reinitiation** | | | | | | | | | | |
|  | No | 47.9 (47.3–48.5) | 57.8 (57.2–58.5) | 66.1 (65.4–66.7) |  | | Reference | | Reference | |
|  | Yes | 43.5 (41.3–45.7) | 53.6 (51.3–55.8) | 63.7 (61.1–66.1) |  | | 0.91 (0.85–0.96) | | 1.05 (0.83–1.33) | |
| **Number of non-diabetic medications used in the 6 months before reinitiation** | | | | | | | | | | |
|  | 0–1 | 57.4 (55.8–59.1) | 66.1 (64.4–67.7) | 72.7 (71.0–74.3) |  | | Reference | | Reference | |
|  | 2–3 | 52.6 (51.4–53.8) | 63.0 (61.8–64.2) | 70.5 (69.2–71.6) |  | | 0.92 (0.88–0.96) | | 0.95 (0.90–0.99) | |
|  | 4–5 | 47.9 (46.6–49.1) | 58.0 (56.7–59.2) | 66.2 (64.8–67.5) |  | | 0.88 (0.84–0.92) | | 0.93 (0.89–0.98) | |
|  | 6–7 | 43.4 (41.9–44.9) | 53.5 (52.0–55.0) | 62.8 (61.1–64.4) |  | | 0.79 (0.75–0.83) | | 0.86 (0.82–0.91) | |
|  | 8–9 | 40.7 (38.8–42.7) | 50.2 (48.1–52.2) | 60.5 (58.2–62.7) |  | | 0.77 (0.72–0.82) | | 0.85 (0.80–0.91) | |
|  | 10–19 | 37.4 (35.8–39.1) | 47.5 (45.7–49.3) | 57.4 (55.4–59.3) |  | | 0.72 (0.68–0.76) | | 0.82 (0.77–0.88) | |
|  | >20 | 39.7 (32.8–46.5) | 47.7 (40.3–54.7) | 57.0 (48.5–64.6) |  | | 0.72 (0.57–0.92) | | 0.90 (0.71–1.14) | |
| **Glucose test in the 6 months before reinitiation ^§^** | | | | | | | | | | |
|  | No | 52.6 (51.6–53.7) | 62.5 (61.5–63.5) | 70.5 (69.4–71.5) |  | | Reference | | Reference | |
|  | Yes | 45.0 (44.3–45.7) | 54.9 (54.2–55.7) | 63.5 (62.7–64.3) |  | | 0.84 (0.81–0.87) | | 0.91 (0.87–0.95) | |
| **Urinary albumin/creatinine ratio test in the 6 months before reinitiation** | | | | | | | | | | |
|  | No | 49.3 (48.5–50.1) | 59.3 (58.5–60.1) | 67.6 (66.7–68.4) |  | | Reference | | Reference | |
|  | Yes | 45.3 (44.4–46.2) | 55.2 (54.2–56.1) | 63.6 (62.6–64.6) |  | | 0.92 (0.89–0.94) | | 0.94 (0.91–0.97) | |

^*^ Adjusted for all other covariates in the table.

^†^ In the New Zealand healthcare system, people can record up–six ethnic groups. For statistical purposes, each individual can be allocated to a single ethnic group using a prioritisation algorithm [32]. The MELAA group (Middle Eastern, Latin American, African) was included in Other.

^‡^ Of the 28,621 people included in this analysis, ethnicity was unknown for 984.

^§^ Record of a laboratory test in the ‘blood glucose’ category (includes HbA1c, fructosamine, glucose tolerance, and serum glucose tests).
